# Effectiveness comparison of an indicated child-centered group prevention program for disruptive behavior problems in children with vs. without co-occurring emotional problems

**DOI:** 10.1186/s40359-025-03392-7

**Published:** 2025-09-08

**Authors:** Cornelia Beate Siegmund, Julia Zink, Patricia Theresa Porst, Max Weniger, Maria McDonald, Susanne Knappe, Veit Roessner, Katja Beesdo-Baum

**Affiliations:** 1 Behavioral Epidemiology, Institute of Clinical Psychology and Psychotherapy, TUD Dresden University of Technology, Chemnitzer Straße 46, 01187 Dresden, Germany; 2 Department of Child and Adolescent Psychiatry, Faculty of Medicine, TUD Dresden University of Technology, Dresden, Germany; 3 German Center for Child and Adolescent Health (DZKJ), partner site Leipzig/Dresden, Dresden, Germany; 4 Evangelische Hochschule Dresden (ehs), University of Applied Sciences for Social Work, Education and Nursing, Dresden, Germany

**Keywords:** Children, Disruptive behavior, Emotional problems, Anxiety, Indicated prevention

## Abstract

**Background:**

Disruptive behavior and emotional problems – especially anxiety – are common in children and frequently co-occur. However, the role of co-occurring emotional problems in disruptive behavior intervention response is unclear. This study aimed to compare the effectiveness of an indicated prevention program in children with disruptive behavior problems with vs. without co-occurring emotional problems.

**Methods:**

Children were screened for disruptive behavior and emotional problems during routine health check-ups and – if indicated – were offered a child-centered group prevention program. For children with disruptive behavior the “Baghira training” was administered. Training effectiveness was compared between participating children with vs. without emotional problems regarding disruptive behavior and emotional problems and anxiety in particular. Outcomes were measured before and after the training, and at six months follow-up using linear mixed effect model regression analyses.

**Results:**

Overall, regarding disruptive behavior, children with and without co-occurring emotional problems profited equally from the Baghira training and training effectiveness was independent of the pre-training level of anxiety. However, there were few indications for greater disruptive behavior symptom reduction in children with co-occurring emotional problems. Overall, the Baghira training had little to no effect on the examined emotional problems/anxiety measures, except the Strengths and Difficulties Questionnaire emotional problems score strongly decreased in children with co-occurring emotional problems.

**Conclusions:**

The effectiveness of the Baghira training is not negatively affected by co-occurring emotional problems/anxiety. However, as emotional problems/anxiety do not simultaneously improve, an additional training targeting these problems for respective children seems necessary.

## Background

Disruptive behavior (DB) disorders such as Oppositional Defiant Disorder (ODD), Conduct Disorder, Intermittent Explosive Disorder and – in a broader sense –Attention Deficit Hyperactivity Disorder are besides anxiety disorders among the most frequent mental disorders before adulthood [1, 2]. Anxiety and DB disorders both usually develop and firstly manifest in childhood or early adolescence [3–5]. Co-occurring disorders are common to occur alongside DB disorders, and apart from co-occurrence between DB disorders, previous studies evidenced that anxiety and depression (together also referred to as internalizing/emotional disorders) frequently co-occur [5–7]. As anxiety disorders usually emerge earlier than depression [8], the anxiety – DB co-occurrence appears particularly crucial in childhood. Wagner et al. [7] reported that 52.6% of the 10–18 year olds with a DB disorder also met criteria for an anxiety disorder. Shared factors may explain this co-occurrence including genetic predisposition, environmental factors like parent-child interactions and peer processes as well as individual predispositions like child’s temperament or biases in information processing and attention, but there are still many etiological questions unresolved [9–11]. For example, the p-factor as one general factor for psychopathology [12] has been suggested to explain this co-occurrence of different mental health dimensions.

Studies show that childhood mental disorders increase the risk for mental disorders in adulthood [13, 14]. Moreover, subthreshold mental disorders where at least one diagnostic criterion is missing or incomplete, increase the risk for further mental health problems and full-blown mental disorders later in life [15, 16]. Research also indicates that DB and anxiety problems in early life have a negative impact on the further development, physical health, interpersonal relations and professional life, and cause high costs in health and social services [17–19]. Hence, close attention should be paid to arising mental health problems and subthreshold mental disorders in childhood. Furthermore, early (preventive) intervention measures are important to support and strengthen mental health and to counteract an adverse development over the lifespan at an early stage. Literature shows that indicated prevention programs are able to decrease symptom severity [20, 21] and even reduce the incidence of new onset cases [22]. Further, research demonstrates that a number of indicated prevention programs for different mental disorders are cost effective [18, 23, 24]. There is also evidence of the efficacy of different indicated prevention programs for DB problems [21]. Smedler et al. [21] reviewed randomized controlled trials and found that indicated prevention programs such as Family Check-Up and Coping Power are able to reduce DB problems, and also have long-term effects. However, overall they observed an inconsistent picture regarding the efficacy of different prevention programs and highlighted the general need for studies examining indicated DB prevention. So far, we have to additionally rely on (early) intervention studies in subjects fulfilling all diagnosis criteria as a proxy for the further deliberations, especially when considering child-centered interventions. Wettach et al. [25] conducted the child-centered Baghira group training [26] in combination with the Triple P-parent training [27] with children with ODD. They found a reduction of DB in the treatment group compared to the control group for different parent report measures. These effects remained stable over the 6-month follow-up period.

As stated earlier, co-occurrence is frequent [7]. Moreover, co-occurring mental health problems likely chronify and are associated with greater severity and impairment [28–30]. Therefore, it appears important to understand the impact of co-occurring mental health problems on prevention and intervention effectiveness. However, research on the role of co-occurring conditions on intervention effectiveness is inconsistent. Some studies reported a negative impact on the intervention outcome [31, 32], some showed no impact [32–34] and others even found a positive impact of co-occurrence [35]. The direction of the effect seems to differ depending on the examined main disorder, co-occurring disorder, intervention target group, setting and measurement time points. Considering DB and co-occurring anxiety, Chase et al. [34] for example found that children with ODD and co-occurring separation anxiety disorder profited from the parent-child interaction therapy (PCIT) as well as children without co-occurring anxiety. However, evidence on the role of co-occurring anxiety in children with DB problems on the effectiveness of an indicated child-centered group-program aiming at DB problems is lacking despite its relevance to tailor prevention and early intervention practice. For example, if children with co-occurring anxiety show less improvement from a respective intervention, these children with co-occurring conditions might need to be identified and treated differently. Co-occurring (untreated) anxiety could interfere with processing and practicing the content for DB problems, thereby limiting effectiveness of the intervention. Anxiety symptoms have been linked to impairments in attentional control and working memory which are greater the higher the anxiety severity [36, 37]. Even though studies show that early intervention programs reduce DB despite co-occurring anxiety [34] it is still an open question whether intervention effects on DB are similar or different in children with or without co-occurring anxiety.

Additionally, an intervention targeting DB could also positively affect co-occurring anxiety, since a DB intervention might address factors that are shared with anxiety like parent-child interaction, peer processes and biases in information processing and attentional control. A recent meta-analysis found positive effects of parenting programs for children’s conduct problems on co-occurring emotional problems, however, the effects were small and not stable over time [38]. Also, Chase et al. [34] found that children with ODD also significantly decreased in their co-occurring anxiety symptoms following PCIT. In contrast, Seeley et al. [39] reported no significant change in the internalizing symptoms in preschoolers with DB and at risk for an anxiety disorder following the First Step to Success Intervention, a school- and parent-based program. Vice versa, there is evidence that interventions targeting anxiety are also able to reduce the symptomatology of the co-occurring DB problems [33, 40]. For example, Levy et al. [40] found that a program targeting anxiety reduced DB problems in children with anxiety and co-occurring aggression as good as the same program with additional content for managing child anger and aggression. However, evidence concerning child-based DB intervention effectiveness on co-occurring anxiety is lacking. Therefore, further research should also be drawn to this issue.

To address these questions, data of a population-based study in which children were screened for DB and emotional problems during routine health check-ups at their pediatrician’s office was used. Children screened positively for DB problems were recommended to participate in an indicated prevention program aiming at DB; children with a current diagnosis of DB or emotional/anxiety disorders were excluded from program participation, and instead referred to local health care services for further evaluation. According to the queries delineated above, the current paper addresses the following hypotheses: (1) Children with co-occurring emotional problems show less improvement in DB scores compared to children with DB problems only and the higher the anxiety, the smaller the improvement. However, we expect improvement in DB measures in both children with and without co-occurring emotional problems. (2) The indicated prevention program addressing DB also reduces symptomatology of emotional problems and anxiety in particular. We expect greater improvement in subjects with co-occurring emotional problems since children with DB problems only have low emotional problems scores prior to program participation and possible bottom effects might prevent great improvement in this group.

## Methods

### Study procedure

#### Screening and recruitment

The present study uses data from the PROMPt project [41] – a prospective non-randomized screening and indicated prevention implementation study that took place from 10/2018 until 09/2022. Parents in the municipal area of Dresden, Germany, reported about emotional and DB problems of their child using the Strengths and Difficulties Questionnaire (SDQ; [42, 43]), at the regular routine pediatric health check-up U9-U11^1^ (usually U9 for 5 year olds, U10 for 7 to 8 year olds, U11 for 9 to 10 year olds). The pediatricians gave feedback to the parents based on the results of the screening questionnaire and their clinical expertise and, if indicated, recommended participation in an indicated prevention program provided by the study team. These were the group program “Mutig werden mit Til Tiger” (Becoming brave with Til Tiger, Tiger training; [44]) for shy and anxious children and the adaptation of the Baghira group training by Aebi et al. [26] for children with DB problems (Baghira training).

Families interested in participating in one of the two trainings were invited to an on-site initial interview with a project member (psychologist) at the TUD Dresden University of Technology (TUD) to determine if the child would benefit from the training and inclusion criteria were fulfilled. Exclusion criteria for training participation were a diagnosed DB or emotional/internalizing ICD-10 mental disorder or unstable medication in the last 6 months, a current psychotherapeutic treatment, and acute endangerment of self or others.

Additionally, children of families who contacted the study team directly were screened with the SDQ and evaluated by the study team at TUD if there was no regular U9/U10/U11 coming up soon or the child had private health insurance. If the child was eligible for participating in the training after the personal evaluation appointment it was assigned to a training group with similar aged children (± 1 year) by the study team. Children with problems in both DB and anxiety were eligible for the participation in both trainings. In these cases, a participative decision was derived between study team member, parents and child, if applicable, which training was more appropriate. In six cases, both trainings were conducted consecutively (three in this analysis sample).

#### Assessments

Besides the initial SDQ assessment and prevention indication evaluation (screening), assessments of participants with project-specific questionnaires took place before (T0; separate appointment few days/weeks before the first training session) and after (T1; separate appointment few days/weeks after the last training session) completing the training and about 6-months after training (T2; approx. 12 months after screening, approx. 6 months after post). In the following, screening and T0 will together be considered “pre”, as they refer to the assessments before training. “Post” will be used for T1 and “follow-up” for T2. All questionnaire measures considered in the manuscript were administered at all three assessment time points: at pre and post via tablet (besides SDQ screening via paper-pencil) and online at follow-up.

The study procedures were explained in written and verbally. All legal guardians gave their written informed consent and children their verbal consent for participation in the training and the questionnaire assessments prior to the first assessment/training session. Families received 10€ for each assessment (pre, post, follow-up) they fully filled in.

### Measures

*Strengths and Difficulties Questionnaire (SDQ)*. The German version of the SDQ for 4- to 17-year-olds [42, 43] was used as the screening instrument. It consists of 25 items rated on a 0- to 2-point response-scale (0 = not true, 1 = somewhat true, 2 = certainly true). The five sub-scales emotional problems, conduct problems, hyperactivity/inattention, peer relationship problems and prosocial behavior each consist of 5 of the 25 items. The sum scores for the four problem scales were calculated with higher scores indicating greater problems. Scores of the sub-scales were calculated when no more than 2 items were missing [corresponding to 45]. Missing values were imputed by the child’s mean score of the respective sub-scale. The sum scores can be categorized as normal, borderline, or abnormal based on cut-off scores as follows: emotional problems 0–3, 4, 5–10; conduct problems 0–2, 3, 4–10; hyperactivity/inattention 0–5, 6, 7–10; peer relationship problems 0–2, 3, 4–10 [42]. For the PROMPt project, the abnormal cut-off scores were slightly modified to reach more children with potential prevention indication (emotional problem scale 0–3, 4–6, 7–10; conduct problem scale 0–2, 3–5, 6–10). Children with at least borderline scores at the emotional problem scale were considered potentially eligible for the Tiger training. Children with at least borderline scores at the conduct problem scale were considered potentially eligible for the Baghira training. The sub-scales of the SDQ have moderate to satisfactory internal consistency and test-retest-reliability for parent ratings [46].

*Child Behavior Checklist (CBCL)*. We administered the 33 items of the externalizing behavior scale of the parent report version of the CBCL for 4- to 18 year-olds [47, 48] consisting of 20 items of the aggressive behavior sub-scale and 13 items of the delinquent behavior sub-scale. Answers were given on a 0- to 2-point response-scale (0 = not true, 1 = somewhat/sometimes true, 2 = very true or often true). Sum scores of the two sub-scales for aggressive and delinquent behavior were calculated with higher scores indicating more severe symptomatology. Since a wording error occurred for item number 2 of the aggressive behavior sub-scale (item 16 of total CBCL), this item was treated as a missing value and imputed by the sub-scale’s mean score. The delinquent behavior-scale item “Truanting school” was only administered to school children and otherwise rated as 0 = “not true”. Mean scores of the sub-scales were calculated if no more than 6.9% of the items were missing [48]. Therefore, no real missing values were allowed for calculation. The used sub-scales aggressive and delinquent behavior have satisfactory to good internal consistency and the whole CBCL problem score and the externalizing problem score show good test-retest-reliability [47, 49].

*Diagnostic System for Psychiatric Disorders III (DISYPS) Parent Rating Scale for Oppositional Defiant Disorder and Conduct Disorder [German: Fremdbeurteilungsbogen für Störungen des Sozialverhaltens] (FBB-SSV)*. The FBB-SSV of the DISYPS [50] was administered to the parents and consists of six sections with items rated on a 0- to 3-point response-scale (0 = not true, 1 = somewhat true, 2 = broadly true, 3 = very true). Section A contains 8 items about oppositional behavior. Section B asks 16 questions concerning aggressive-dissocial behavior where 10 items are only to be answered for children 11 years or older and were therefore not administered in this study. Further, item B1 asking about sibling conflict was not displayed if the parent indicated earlier that the index child had “no” siblings and treated as 0 for score calculation. Section C contains 11 items about limited prosocial emotionality and section D consists of three items that build the sub-scale disruptive affect regulation disorder and irritability in combination with the first three items of section A. Both sections C and D are supposed to be asked only when the child is six years or older. Hence, the sub-scores limited prosocial emotionality and disruptive affect regulation disorder and irritability were only calculated for children aged 6 years or older at the first assessment. Sections F and K sub-scales were not examined in this study. Sum scores were calculated when no more than 10% of the items were real missing values (items that should have been answered). These were conservatively calculated as 0 “not true” according to the questionnaire instruction [50]. Higher scores of the examined sub-scales indicate more severe symptomatology. The sub-scales of the FBB-SSV have moderate to good internal consistency [50]. Test-retest-reliability was not available for the questionnaire.

*Screen for Child Anxiety Related Emotional Disorders (SCARED)*. To assess anxiety the German version of the SCARED was used [51–53]. The questionnaire consists of 41 items rated on a 0- to 2-point response-scale (0 = not true or hardly ever true, 1 = somewhat true or sometimes true, 2 = very true or often true) and has good internal consistency and test-retest-reliability [51, 53]. Sum scores for the sub-scales panic/somatic (13 items), general anxiety (9 items), separation anxiety (8 items), social phobia (7 items) and school phobia (4 items) where calculated with higher scores indicating higher anxiety severity. Missing values were not imputed because all subjects of the used sub-sample either had all items properly filled in or more than 30% missing, which would have been the maximum for imputation [corresponding to 45].

### Baghira training

The Baghira training is based on the Baghira group training for children with oppositional and aggressive behavior by Aebi et al. [26], which is evaluated to be effective in reducing DB in children with ODD in combination with the Triple P-parent training in a quasi-experimental design [25]. Evaluations of the Baghira group training alone as well as the adapted Baghira training used in this study are currently pending. The training consisted of nine weekly 90 min group sessions of three to five children (M = 4.24, SD = 0.45; ideal group size of six was reduced due to the Covid-19 pandemic). It was conducted at TUD by a certified trainer (M.Sc. Psychology study team member or psychology master student). In sum, 19 certified trainers conducted a Baghira training course. During the training, the children learned strategies to control their anger and appropriately resolve conflicts. All training sessions followed the same structure: First, after repeating the group rules, a short fantasy journey for relaxation was conducted. Second, the content of the last session was briefly repeated and the homework discussed. Then the current topic was presented and the desired behavior was practiced in role plays and the new homework assignment was explained. At the end, each child assessed themselves regarding their rule compliance and received the trainer’s feedback. Finally, children displayed their current mood using black or white beads. Additionally, desired behavior was encouraged through a reward program and the children were asked to practice during the week what they had learned in the last session. The topics of the sessions contained emotion and self-awareness, dealing with anger and aggression, impulse control, conflict- and problem-solving, empathy, change of perspective and giving feedback. It uses common cognitive-behavioral methods such as contingency management, observational learning, self-verbalization and cognitive restructuring, which are also used in cognitive-behavioral therapy with children suffering from disruptive behavior disorders. The Baghira training was originally designed for 8–13 year olds but was slightly modified for the study to also fit younger kids (5–10 years). For example, the sessions were reduced from 120 min to 90 min, including a short break. Furthermore, we added a 90-minute information evening for parents conducted concurrently to one of the training sessions. In this, parents were given information and advice on appropriately reacting in everyday situations and helping the child handle anger and frustration.

### Analysis sample

**Fig. 1:**
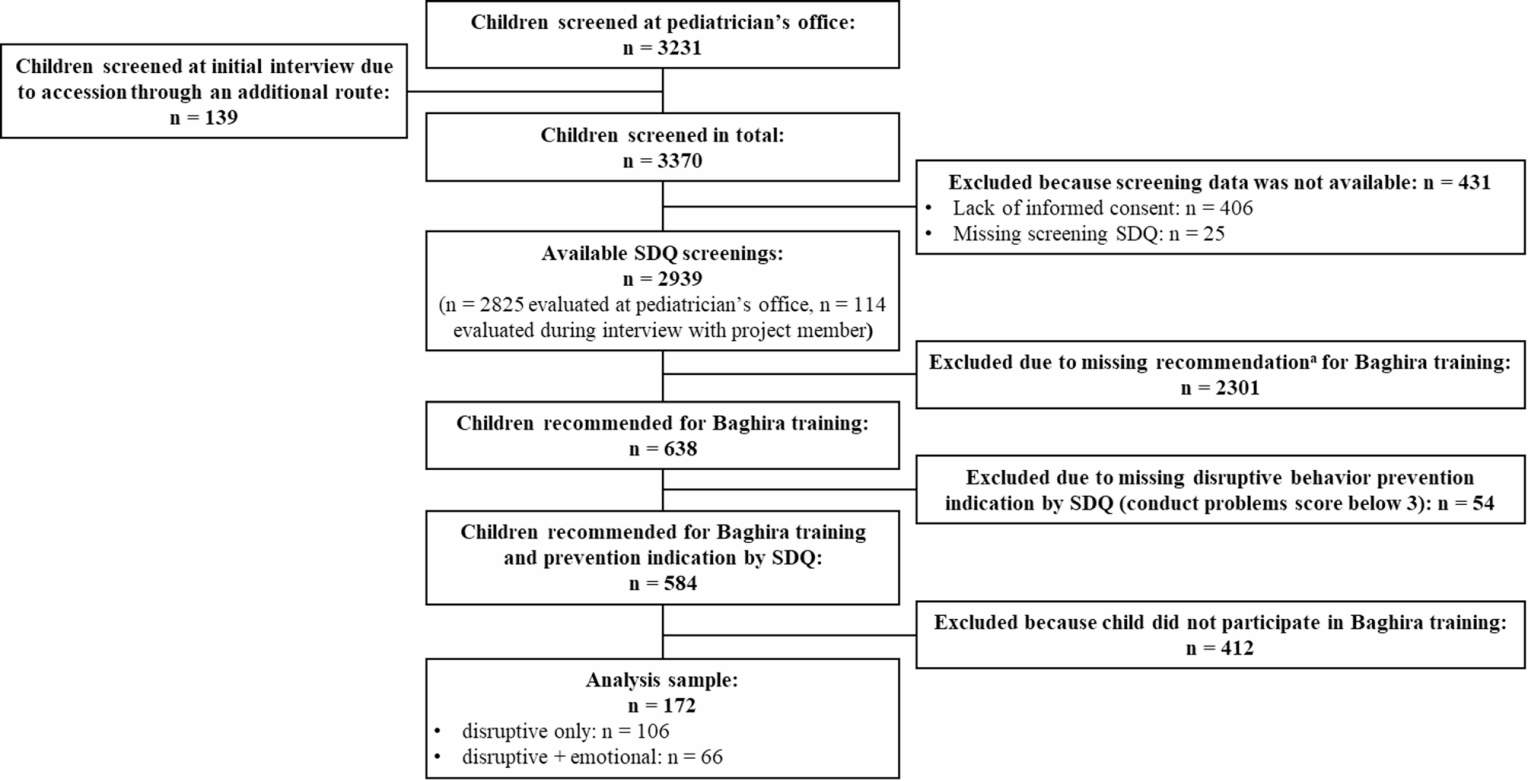
Flowchart of the analysis sample. ^a^ recommendation for participation in Baghira training by the pediatrician or at the interview with the project member; n = number of participants; SDQ = Strengths and Difficulties Questionnaire; disruptive only = group of children screened below the borderline SDQ emotional problems cut-off of 4; disruptive + emotional = group of children screened at or above the borderline SDQ emotional problems cut-off of 4

To examine the research questions of this current study, parent-report data from Baghira training participants for whom the DB prevention program was indicated were used. Children who attended the Baghira training but scored below the SDQ conduct problem borderline cut-off of 3 were excluded from this study’s analysis sample (*n* = 15). This was done to ensure that examined children had a confirmed prevention indication by questionnaire score and a measurable level of DB to detect the hypothesized symptom reduction through training. Children who participated in the Baghira training scoring above the abnormal cut-off of 6 were included in the analysis sample when evaluated as indicated (*n* = 33 in final analysis sample). Comprehensive information on the whole sample of the PROMPt project can be found in Weniger et al. [54]. A flowchart of the analysis sample is illustrated in Fig. 1. Overall, SDQ screening and consent was available for *n* = 2939 children (*n* = 2825 evaluated at pediatrician’s office, *n* = 114 directly evaluated during interview with project member). Of these, *n* = 638 children were recommended to participate in the Baghira training, of whom *n* = 584 scored above or at the borderline cut-off of the SDQ sub-scale conduct problems (*n* = 513 scored between 3 and 5 and *n* = 71 between 6 and 10). Finally, *n* = 172 children attended the Baghira training, with *n* = 168 completing and *n* = 4 quitting the training. The children who completed the Baghira training participated in 6–9 of 9 sessions (M = 8.38, SD = 0.76) and the children who quitted participated in 2–6 of 9 training sessions (M = 4.00, SD = 1.83). Furthermore *n* = 3 of the 172 children also participated in the Tiger training – the indicated prevention program for shy and anxious children – after completing the Baghira training between the post and follow-up assessments (for one of these children the follow-up data are missing). For the purpose of the current analyses, the total sample of *n* = 172 children (41 female, 131 male) was divided into two groups based on their SDQ emotional problem score: *n* = 106 children (22 female, 84 male) scored below the borderline cut-off of 4 [“disruptive only” group] and *n* = 66 children (19 female, 47 male) scored at or above it [“disruptive + emotional” group]. Children of this analysis sample were between 4 and 11 years old at screening (data from *n* = 170, M = 6.8, SD = 1.87) and between 5 and 12 years old at follow-up (data from *n* = 127, M = 7.91, SD = 1.89).

### Data analysis

The specific hypotheses of this work relating the impact of co-occurring anxiety were secondary analyses to the PROMPt project. Statistical analyses were carried out using the software STATA version 17 [55]. Descriptive statistics (mean, M; standard deviation, SD; percent, %) were calculated regarding socio-demographic and other sample characteristics, including the pre-training questionnaire scores for both the total sample and the groups “disruptive only” and “disruptive + emotional”. To examine whether the two groups differ, Chi^2^-tests for categorical and t-tests for metric variables were conducted. To compare the effectiveness of the Baghira training, linear mixed effect model regressions combined with the robust Huber/White/sandwich estimator were calculated for DB and emotional problems/anxiety questionnaire scores focusing on the interactions pre to post x group and pre to follow-up x group and the main effects pre to post and pre to follow-up. To analyze whether children show less reduction in DB the higher the pre-training SCARED total anxiety score (pre-anxiety), linear mixed effect model regressions combined with the robust Huber/White/sandwich estimator were calculated for the DB questionnaire scores focusing on the interactions pre to post x pre-anxiety and pre to follow-up x pre-anxiety and the main effects pre to post and pre to follow-up. For graphic presentation of these interaction effects, children were categorized based on their pre-anxiety: Children who were rated more than one SD below/above the mean pre-anxiety score were categorized as low/high anxious, respectively. Children scoring in between were categorized as moderate anxious. In all calculated linear mixed effect models, subjects were included as random effect to account for inter-individual differences between subjects when comparing the analysis groups. Children’s age and sex, as well as number of participated sessions and timespan of screening to post and of screening to follow-up were taken into account as covariates. Before calculating, the mixed effect models questionnaire scores were standardized by the pooled standard deviation by group at pre-training for better comparison of the outcomes and interpreting beta coefficients as effect sizes. As mixed effect model regressions are robust against missing data [56] and the proportion of missing sum scores at pre and post was low (0-5.8% at pre, 6.4–8.7% at post, 26.7–29.1% at follow-up depending on questionnaire), we decided against imputation procedures. The alpha level for all analyses was a priori set at 0.05. Alpha adjustment for multiple testing was omitted to be sensitive to effectiveness differences as the hypotheses are built on an exploratory approach due to inconsistent results in literature [e.g. 32, 34, 39]. All the above mentioned analyses were additionally performed without the three children who participated in the Tiger training after the Baghira training. These analyses revealed similar results as the results including these children reported below. In addition, it was calculated in how many children with and without co-occurring emotional problems the SDQ conduct problems and SDQ emotional problems score dropped below the prevention indication cut-off of 3 respectively 4 through training.

## Results

### Sample characteristics

The characteristics of the whole analysis sample (*n* = 172) and comparison between the two groups “disruptive only” (*n* = 106) and “disruptive + emotional” (*n* = 66) can be found in Table 1. Overall, the sample consists of three times as many male as female children with no difference in sex distribution between the groups. On average, the children in the “disruptive only” group were younger than the children in the “disruptive + emotional” group (*p* = .018). The pre-scores of DB measures did not differ between groups, except for the DISYPS disruptive affect regulation disorder and irritability sub-scale which was lower in the “disruptive only” group vs. “disruptive + emotional” group (*p* = .030). As expected, all pre-scores of emotional problems and anxiety measures were higher in the “disruptive + emotional” group vs. the “disruptive only” group (all *p* < .004). None of the other characteristics differed between groups.

**Table 1:** Sample characteristics

	Total Sample (*n* = 172)		Disruptive Only (*n* = 106)		Disruptive + Emotional (*n* = 66)		Group Comparison	
	*n*	% / M (SD)	*n*	% / M (SD)	*n*	% / M (SD)	Chi2 / t-test (df)	*p*-value
**Sex Child**								
female	41	23.8	22	20.8	19	28.8	1.45 (1)	0.229
male	131	76.2	84	79.2	47	71.2		
**Age Child at Screening**	170	6.80 (1.87)	105	6.53 (1.80)	65	7.23 (1.92)	-2.4 (168)	0.018
**Nationality Child**								
German	132	99.2	86	100.0	46	97.9	1.84 (1)	0.175
Other	1	0.8	0	0.0	1	2.1		
**Nationality Mother**								
German	130	97.7	84	97.7	46	97.9	0.01 (1)	0.941
Other	3	2.3	2	2.3	1	2.1		
**Nationality Father**								
German	117	95.9	78	96.3	39	95.1	0.10 (1)	0.757
Other	5	4.1	3	3.7	2	4.9		
**Parents’ Monthly Net Income**								
less than 1000€	4	3.2	3	3.8	1	2.2	4.82 (4)	0.307
1000–2000€	20	16.1	9	11.4	11	24.4		
2000–3000€	24	19.4	16	20.3	8	17.8		
3000–4000€	42	33.9	26	32.9	16	35.6		
more than 4000€	34	27.4	25	31.6	9	20.0		
**Timespan Screening to Post in Days**	164	219.61 (77.64)	101	224.87 (80.23)	63	211.17 (73.12)	1.10 (162)	0.273
**Timespan Post to Follow-up in Days**	125	190.14 (55.15)	79	188.03 (55.27)	46	193.76 (55.35)	-0.56 (123)	0.577
**Timespan Screening to Follow-up in Days**	127	403.09 (52.22)	80	404.52 (54.9)	47	400.64 (47.78)	0.40 (125)	0.687
**Training Completer/Quitter**								
Completer	168	97.7	104	98.1	64	97.0	0.23 (1)	0.628
Quitter	4	2.3	2	1.9	2	3.0		
**Number Participated Sessions in Baghira Training (maximum 9)**								
Total	172	8.27 (1.03)	106	8.28 (1.03)	66	8.26 (1.03)	0.16 (170)	0.875
Completer	168	8.38 (0.76)	104	8.38 (0.77)	64	8.38 (0.75)	0.00 (166)	1.000
Quitter	4	4.00 (1.83)	2	3.50 (2.12)	2	4.5 (2.12)	-0.47 (2)	0.684
**Participation in Tiger Training after Baghira Training**								
No	169	98.3	105	99.1	64	97.0	1.03 (1)	0.309
Yes	3	1.7	1	0.9	2	3.0		
**Measures of Disruptive Behavior Problems Pre-Training**								
SDQ Conduct Problems^a^	172	4.59 (1.36)	106	4.44 (1.21)	66	4.83 (1.55)	-1.85 (170)	0.066
SDQ Hyperactivity/Inattention	172	5.74 (2.37)	106	5.53 (2.42)	66	6.08 (2.27)	-1.50 (170)	0.136
CBCL Aggressive Behavior	165	17.49 (6.17)	100	17.42 (6.21)	65	17.59 (6.17)	-0.17 (163)	0.867
CBCL Delinquent Behavior	165	3.94 (2.38)	100	3.92 (2.41)	65	3.97 (2.35)	-0.13 (163)	0.897
DISYPS Oppositional Behavior	165	12.51 (4.72)	101	12.04 (4.93)	64	13.25 (4.31)	-1.61 (163)	0.109
DISYPS Aggressive-Dissocial Behavior	165	3.11 (2.40)	101	3.26 (2.36)	64	2.88 (2.47)	1.00 (163)	0.321
DISYPS Limited Prosocial Emotionality^b^	97	7.42 (5.75)	52	7.13 (5.51)	45	7.76 (6.05)	-0.53 (95)	0.598
DISYPS Disruptive Affect Regulation Disorder and Irritability^b^	97	8.56 (3.68)	52	7.81 (3.66)	45	9.42 (3.55)	-2.20 (95)	0.030
**Measures of Emotional Problems/Anxiety Pre-Training**								
SDQ Emotional Problems^a^	172	3.08 (2.31)	106	1.58 (1.07)	66	5.48 (1.63)	-18.97 (170)	< 0.001
SDQ Peer Relationship Problems	172	2.54 (2.21)	106	2.14 (1.90)	66	3.17 (2.51)	-3.03 (170)	0.003
SCARED Total Anxiety	166	16.55 (10.71)	101	11.85 (7.39)	65	23.86 (11.02)	-8.41 (164)	< 0.001
SCARED Panic/Somatic	166	1.89 (2.93)	101	0.94 (1.42)	65	3.37 (3.91)	-5.69 (164)	< 0.001
SCARED General Anxiety	166	5.13 (3.79)	101	3.7 (2.95)	65	7.35 (3.91)	-6.84 (164)	< 0.001
SCARED Separation Anxiety	166	4.22 (3.15)	101	3.29 (2.79)	65	5.68 (3.15)	-5.12 (164)	< 0.001
SCARED Social Phobia	166	4.57 (3.67)	101	3.47 (3.10)	65	6.28 (3.84)	-5.19 (164)	< 0.001
SCARED School Phobia	166	0.74 (1.19)	101	0.46 (0.82)	65	1.18 (1.51)	-4.03 (164)	< 0.001

Note. M = mean; SD = standard deviation; df = degrees of freedom; *p* = *p*-value; n = sample size/number of participants; Tiger Training = indicated group program for shy and anxious children “Mutig werden mit Til Tiger” (Becoming brave with Til Tiger); differences in sample size are due to missing or partially filled in questionnaires; ^a^ questionnaire scores used for sampling; ^b^ questionnaire scores not calculated for under 6-year olds

### Effectiveness on disruptive behavior

**Fig. 2:**
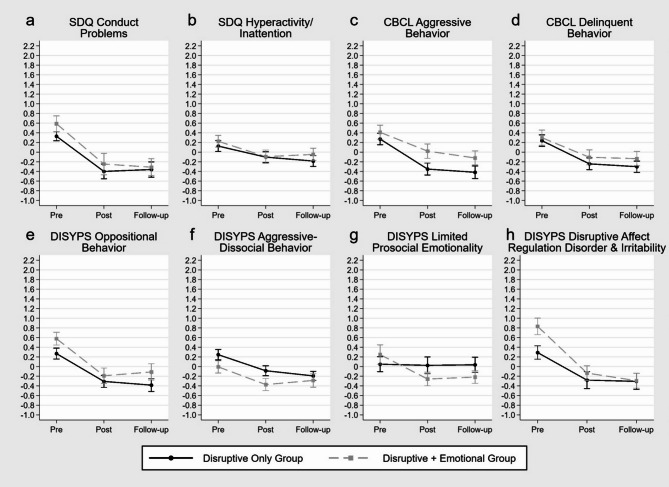
Estimated marginal means and standard errors based on the calculated mixed effect models for standardized disruptive behavior measures by group over the three assessments pre, post and follow-up

*Effectiveness by Group*. The results of the respective linear mixed effect models are shown in Table 2; Fig. 2. They demonstrated differences in improvements in DB scores by group only for the DISYPS limited prosocial emotionality score (Fig. 2g) and the DISYPS disruptive affect regulation disorder and irritability score (Fig. 2h). The former exhibited significant interactions for each pre to post (*p* = .020) and pre to follow-up (*p* = .021) by group. For the latter, only the interaction pre to follow-up by group was significant (*p* = .024). This indicates greater symptom reduction in these two scores through training in children with co-occurring emotional problems compared to children with DB problems only. Actually, the “disruptive only” group did not show a change in the DISYPS limited prosocial emotionality score through training. All of the DB measures not showing any interaction demonstrated significant main effects pre to post and pre to follow-up. This indicates improvements in the DB problems through training for the whole sample measurable at both post-training and follow-up.

**Table 2:** Results of main effects and interaction effects of the calculated linear mixed effect models of disruptive behavior measures by group

	Pre to Post				Pre to Follow-up				Group				Pre to Post x Group				Pre to Follow-up x Group			
	β	95% CI		*p*	β	95% CI		*P*	β	95% CI		*p*	β	95% CI		*p*	β	95% CI		*p*
SDQ Conduct Problems^a^	-0.73	-0.99	-0.46	< 0.001	-0.69	-0.96	-0.42	< 0.001	0.26	-0.12	0.64	0.180	-0.11	-0.56	0.35	0.646	-0.21	-0.62	0.20	0.313
SDQ Hyperactivity/ Inattention	-0.23	-0.44	-0.01	0.038	-0.31	-0.51	-0.10	0.003	0.10	-0.23	0.43	0.547	-0.09	-0.43	0.25	0.597	0.04	-0.27	0.35	0.815
CBCL Aggressive Behavior	-0.62	-0.79	-0.45	< 0.001	-0.69	-0.91	-0.46	< 0.001	0.14	-0.23	0.51	0.463	0.23	-0.05	0.51	0.106	0.16	-0.18	0.50	0.361
CBCL Delinquent Behavior	-0.48	-0.69	-0.27	< 0.001	-0.54	-0.77	-0.30	< 0.001	0.06	-0.33	0.46	0.761	0.07	-0.22	0.37	0.622	0.11	-0.21	0.42	0.515
DISYPS Oppositional Behavior	-0.58	-0.79	-0.37	< 0.001	-0.65	-0.89	-0.42	< 0.001	0.31	-0.03	0.65	0.076	-0.19	-0.53	0.15	0.273	-0.04	-0.40	0.33	0.844
DISYPS Aggressive-Dissocial Behavior	-0.33	-0.52	-0.15	< 0.001	-0.44	-0.62	-0.26	< 0.001	-0.25	-0.58	0.08	0.132	-0.03	-0.33	0.26	0.821	0.16	-0.13	0.44	0.282
DISYPS Limited Prosocial Emotionality ^b^	-0.02	-0.29	0.24	0.855	-0.01	-0.26	0.23	0.919	0.20	-0.31	0.71	0.443	-0.48	-0.89	-0.07	0.020	-0.45	-0.84	-0.07	0.021
DISYPS Disruptive Affect Regulation Disorder and Irritability ^b^	-0.57	-0.92	-0.22	0.002	-0.60	-0.90	-0.29	< 0.001	0.54	0.11	0.97	0.013	-0.40	-0.91	0.12	0.129	-0.53	-0.99	-0.07	0.024

Note. β = standardized beta coefficient; CI = confidence interval; *p* = *p*-value; ^a^ questionnaire score used for sampling; ^b^ questionnaire scores not calculated for under 6-year olds

**Fig. 3:**
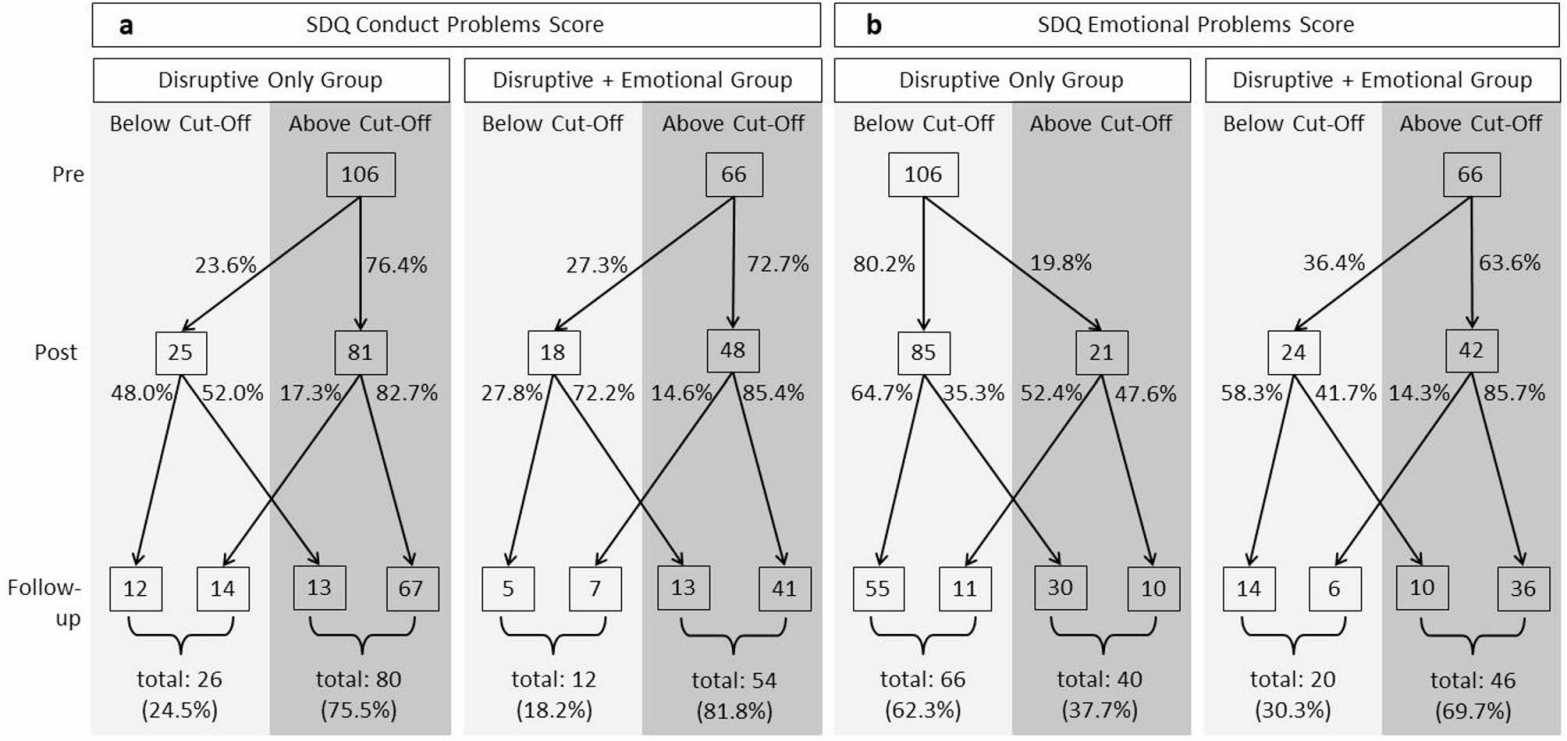
Flowchart of children rated below or above (**a**) the borderline/prevention indication cut-off of 3 regarding disruptive behavior problems measured with the SDQ conduct problems scale and (**b**) the borderline/prevention indication cut-off of 4 regarding emotional problems measured with the SDQ emotional problems scale. Each presented for the two groups “disruptive only” and “disruptive + emotional” over the three assessments pre, post and follow-up

The flowchart in Fig. 3a shows the children of the two analysis groups categorized as below or above the SDQ conduct problems scale borderline cut-off over the three assessments. It shows that in both groups almost the same proportion of children dropped below the cut-off or stayed above it at each post and follow-up. This indicates that children with and without co-occurring emotional problems seem to profit similarly from the Baghira training.

**Fig. 4:**
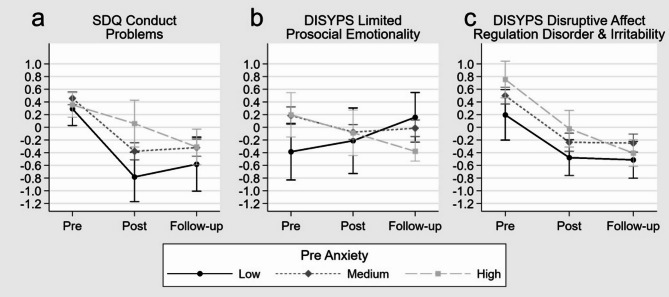
Estimated marginal means and standard errors based on the calculated mixed effect models for standardized disruptive behavior measures by pre-anxiety (SCARED total anxiety score at pre-training) categorized as low, medium and high over the three assessments pre, post and follow-up

*Effectiveness by Pre-Anxiety*. The results of the respective linear mixed effect models are shown in Table 3. For SDQ conduct problems, an interaction effect for pre to post (*p* = .036) but not for pre to follow-up by pre-anxiety emerged. These results indicate that the higher the pre-anxiety, the greater was the reduction of the SDQ conduct problems score from pre to post (Fig. 4a). This effect was also found for the DISYPS limited prosocial emotionality score showing significant interactions for both pre to post (*p* = .014) and pre to follow-up (*p* = .004) by pre-anxiety. Decomposing the interaction of the DISYPS limited prosocial emotionality score revealed that in children with high pre-anxiety, symptom reduction was greater the higher the pre-anxiety. In contrast, children with low pre-anxiety showed a symptom increase in limited prosocial emotionality that was greater the lower the pre-anxiety. Children with medium pre-anxiety showed no change in the DISYPS limited prosocial emotionality score through training (Fig. 4b). The DISYPS disruptive affect regulation disorder and irritability score also demonstrated an interaction effect but only for pre to follow-up by pre-anxiety (*p* = .0498). According to the other measures, it also indicates that children show greater reduction in the DISYPS disruptive affect regulation disorder and irritability score the higher the pre-anxiety (Fig. 4c). All of the DB measures not showing any interaction demonstrated significant main effects pre to post and pre to follow-up except SDQ hyperactivity/inattention. This indicates a reduction of the respective DB problem scores except for the SDQ hyperactivity/inattention score through training for the whole sample measurable at both post and follow-up.

**Table 3:** Results of main effects and interaction effects of the calculated linear mixed effect models of disruptive behavior measures by SCARED total anxiety score pre-training (pre-anxiety)

	Pre to Post				Pre to Follow-up				Pre-Anxiety				Pre to Post x Pre-Anxiety				Pre to Follow-up x Pre-Anxiety			
	β	95% CI		*p*	β	95% CI		*p*	β	95% CI		*p*	β	95% CI		*p*	β	95% CI		*p*
SDQ Conduct Problems^a^	-1.09	-1.43	-0.75	< 0.001	-0.92	-1.30	-0.55	< 0.001	0.00	-0.01	0.02	0.718	0.02	0.00	0.04	0.036	0.01	-0.01	0.03	0.274
SDQ Hyperactivity/Inattention	-0.24	-0.58	0.10	0.169	-0.33	-0.66	0.00	0.050	0.01	0.00	0.02	0.099	0.00	-0.02	0.02	0.855	0.00	-0.02	0.02	0.871
CBCL Aggressive Behavior	-0.59	-0.82	-0.36	< 0.001	-0.70	-1.01	-0.39	< 0.001	0.01	-0.01	0.03	0.243	0.00	-0.01	0.01	0.629	0.00	-0.01	0.02	0.593
CBCL Delinquent Behavior	-0.45	-0.71	-0.19	0.001	-0.53	-0.80	-0.25	< 0.001	0.01	-0.01	0.03	0.213	0.00	-0.01	0.01	0.810	0.00	-0.01	0.01	0.703
DISYPS Oppositional Behavior	-0.62	-0.91	-0.34	< 0.001	-0.70	-1.03	-0.37	< 0.001	0.01	0.00	0.03	0.131	0.00	-0.01	0.01	0.736	0.00	-0.02	0.02	0.757
DISYPS Aggressive-Dissocial Behavior	-0.24	-0.45	-0.02	0.032	-0.26	-0.52	0.00	0.049	0.01	0.00	0.02	0.142	-0.01	-0.02	0.00	0.147	-0.01	-0.02	0.01	0.325
DISYPS Limited Prosocial Emotionality ^b^	0.07	-0.22	0.35	0.641	0.26	-0.06	0.57	0.115	0.02	-0.01	0.04	0.133	-0.02	-0.03	0.00	0.014	-0.02	-0.04	-0.01	0.004
DISYPS Disruptive Affect Regulation Disorder and Irritability ^b^	-0.65	-1.11	-0.20	0.005	-0.49	-0.84	-0.14	0.006	0.02	0.00	0.04	0.036	-0.01	-0.03	0.01	0.559	-0.02	-0.04	0.00	0.0498

Note. β = standardized beta coefficient; CI = confidence interval; *p* = *p*-value; ^a^ questionnaire score used for sampling; ^b^ questionnaire scores not calculated for under 6-year olds

### Effectiveness on emotional problems

**Fig. 5:**
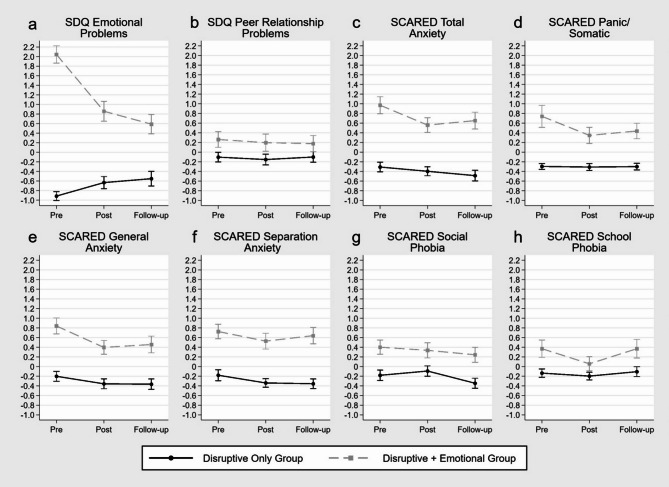
Estimated marginal means and standard errors based on the calculated mixed effect models for standardized emotional problems measures by group over the three assessments pre, post and follow-up

The results of the respective linear mixed effect models are shown in Table 4; Fig. 5. They only demonstrated interaction effects pre to post (*p* < .001) and pre to follow-up (*p* < .001) by group for the SDQ emotional problems score. For SCARED total anxiety (Fig. 5c) and SCARED panic/somatic (Fig. 5d) there were also interaction effects but only for pre to post by group (*p* = .032 and *p* = .035). This indicates greater symptom reduction in the “disruptive + emotional” group through training compared to children with DB problems only in the measures with interaction effects. Evidence against similar symptom reduction in the other measures is lacking. Decomposing the interaction of the SDQ emotional problems score revealed that the “disruptive only” group actually showed a symptom increase, whereas a strong symptom reduction was observed in children with initial co-occurring emotional problems (Fig. 5a). Decomposing the SCARED total anxiety interaction revealed that only the “disruptive + emotional” group showed a symptom reduction, whereas the “disruptive only” group demonstrated no symptom change. Of the emotional problems/anxiety measures not showing any interaction, none showed main effects pre to post and pre to follow-up except SCARED total anxiety pre to follow-up (*p* = .045) and SCARED social phobia pre to follow-up (*p* = .041). This indicates a reduction in the SCARED total anxiety and SCARED social phobia scores from pre to follow-up for the whole sample and no evidence for change through training in the other respective scores.

**Table 4:** Results of main effects and interaction effects of the calculated linear mixed effect models of emotional problems and particular anxiety measures by group

	Pre to Post				Pre to Follow-up				Group				Pre to Post x Group				Pre to Follow-up x Group			
	β	95% CI		*p*	β	95% CI		*p*	β	95% CI		*p*	β	95% CI		*p*	β	95% CI		*p*
SDQ Emotional Problems^a^	0.28	0.04	0.53	0.023	0.36	0.05	0.67	0.021	2.96	2.56	3.35	< 0.001	-1.47	-2.03	-0.90	< 0.001	-1.82	-2.36	-1.28	< 0.001
SDQ Peer Relationship Problems	-0.05	-0.23	0.13	0.583	0.00	-0.16	0.16	0.971	0.37	-0.01	0.74	0.055	-0.02	-0.33	0.30	0.923	-0.09	-0.36	0.19	0.525
SCARED Total Anxiety	-0.09	-0.23	0.06	0.235	-0.18	-0.35	0.00	0.045	1.28	0.88	1.68	< 0.001	-0.32	-0.62	-0.03	0.032	-0.14	-0.53	0.25	0.476
SCARED Panic/Somatic	-0.01	-0.13	0.10	0.834	0.00	-0.13	0.13	0.962	1.04	0.57	1.50	< 0.001	-0.38	-0.74	-0.03	0.035	-0.30	-0.69	0.09	0.133
SCARED General Anxiety	-0.16	-0.34	0.03	0.093	-0.16	-0.34	0.01	0.066	1.04	0.65	1.43	< 0.001	-0.29	-0.60	0.03	0.072	-0.22	-0.59	0.15	0.247
SCARED Separation Anxiety	-0.16	-0.33	0.01	0.064	-0.18	-0.36	0.01	0.064	0.90	0.53	1.27	< 0.001	-0.04	-0.33	0.26	0.797	0.09	-0.30	0.49	0.653
SCARED Social Phobia	0.09	-0.07	0.24	0.264	-0.16	-0.32	-0.01	0.041	0.58	0.22	0.94	0.001	-0.15	-0.47	0.17	0.348	0.01	-0.31	0.33	0.965
SCARED School Phobia	-0.06	-0.26	0.14	0.548	0.03	-0.20	0.26	0.800	0.51	0.12	0.89	0.010	-0.25	-0.58	0.08	0.139	-0.03	-0.49	0.43	0.900

Note. β = standardized beta coefficient; CI = confidence interval; *p* = *p*-value; ^a^ questionnaire score used for sampling

The flowchart in Fig. 3b shows the children of the two analysis groups categorized as below or above the SDQ emotional problems scale borderline cut-off over the three assessments. It shows that most children of the “disruptive only” group stayed below the prevention indication cut-off for emotional problems. However, almost one-fifth rose above it at post and more than one-third scored above it by the time of follow-up. In contrast, most children of the “disruptive + emotional” group stayed above the prevention indication cut-off for emotional problems but about one-third scored below at post and follow-up. This indicates that the Baghira training might be able to offset the prevention indication for emotional problems in children with co-occurring emotional problems. However, this effect seems to be small due to the great number of children staying above the cut-off and even rising above the cut-off without prior prevention indication for emotional problems as seen in the “disruptive only” group.

## Discussion

This intervention study screened children from the general population for DB and emotional problems. It is the first effectiveness comparison of an indicated child-centered group DB prevention program considering the role of co-occurring emotional problems. It contributes information to the question whether early intervention effects on DB differ in children with vs. without co-occurring emotional problems and anxiety in particular.

*Effectiveness on Disruptive Behavior*. Contrary to our hypotheses, the results show no evidence that children with DB and emotional problems profited differently from the indicated prevention program than children with DB only. Also, there is no evidence that symptom reduction in DB through the Baghira training was related to pre-training anxiety levels. Most of the examined measures show no evidence for differences in symptom change between the groups “disruptive only” and “disruptive + emotional” or as a function of pre-anxiety. Therefore, the results are in line with Chase et al. [34] who reported that the PCIT effectiveness was unaffected by co-occurring anxiety and extend their findings to the setting of an indicated child-centered group prevention program addressing DB. Looking at the few interaction effects, results even point into the opposite direction, with greater symptom reduction in children with co-occurring emotional problems or the higher the pre-anxiety. This would be in line withKazdin et al. [35] who found co-occurrence linked to greater therapeutic change. Also, the results ofJarrett et al. [57] indicate that greater depressive symptomatology predicts larger reductions in DB problems following a school-based prevention program aiming at DB problems. However, anxiety was not associated with changes in DB problems in their study either. In the present study beta coefficients were very small for the interaction effects by pre-anxiety, which are accordingly non-relevant. Also, the interaction effect pre to follow-up by pre-anxiety of the DISYPS disruptive affect regulation disorder and irritability score barely reached significance (*p* = .0498) and therefore might be false positive. This further supports the conclusion that the training effectiveness might be independent of anxiety severity. Further, although there were significant interaction effects by group, the overall picture showed no evidence for differences between groups. Also, the interaction effect for the DISYPS disruptive affect regulation disorder and irritability score could be attributed to significant higher pre-training scores in the “disruptive + emotional” group. This might have led to a greater potential in symptom reduction or maybe yield bottom effects in children without co-occurring emotional problems and lower pre-training scores. However, the meta-analysis by McMahon et al. [58] shows mixed evidence on the role of symptom severity on intervention effects, ranging from higher initial severity being associated with greater intervention effects to no effect. In general, it can be concluded that the Baghira training appears to be comparable in terms of effectiveness in children with vs. without co-occurring emotional problems/anxiety. Thus, it seems possible to assign children with DB and co-occurring emotional problems to the same indicated prevention program/early intervention for DB problems as children with DB only. Therefore, concerning DB it seems not necessary to identify children with co-occurring emotional problems as a separate group and treat their DB problems differently. However, asLevy et al. [40] found that treatment of anxiety also reduced DB problems, it could be possible that children with DB and co-occurring emotional problems might exhibit even greater symptom reduction in DB problems when applying additional interventions targeting their emotional problems. This aspect should therefore be examined in future research.

Apart from two exceptions, all measures showed a symptom reduction through training from pre to post and pre to follow-up. Therefore, the results give indications that the Baghira training is able to reduce DB symptoms in general and that it has short- and long-term benefits (at least up to 6 months post training). This is in line with Wettach et al. [25], who examined the Baghira group training in combination with the Triple P-parent training, as well as other studies demonstrating effectiveness of (early) interventions on (arising/subthreshold) DB disorders [34, 39]. Although this is not a randomized efficacy study and has no active control group, results of this quasi-experimental study extend the evidence on the effectiveness of indicated prevention programs for children with DB problems under ecologically valid conditions and add to the inconsistent findings reported by Smedler et al. [21]. The only small to non-existent symptom reduction in the SDQ hyperactivity/inattention score could be due to the content of the Baghira training which does not address hyperactivity and inattention. The missing symptom reduction in the DISYPS limited prosocial emotionality score in the “disruptive only” group must be seen in the light of the symptom change as a function of pre-anxiety. The analysis revealed a symptom reduction in children with high anxiety, no symptom change in children with medium pre-anxiety and a symptom increase in children with low pre-anxiety. Looking at the distribution in the “disruptive only” group: more than 90% of the children were categorized as low and medium anxious. Nevertheless, the question remains unanswered why symptom change in the DISYPS limited prosocial emotionality score depends on the level of pre-anxiety and should be examined in future research.

*Effectiveness on Emotional Problems*. The second aim of this study was to examine whether the indicated group prevention program for DB was also able to reduce emotional problems, especially anxiety. The findings suggest that the program had minimal to no measurable effect on emotional problems/anxiety. Hence, the training content seems to be specific for DB and content addressing shared factors with emotional problems/anxiety seems insufficient for also reducing emotional problems/anxiety. The long-term symptom reduction in the whole sample was only present in two measures (SCARED total anxiety, SCARED social phobia) with *p*-values just below the significance level. Considering that no correction for multiple testing was applied, these main effects might be false positive. Therefore, they do not prove the training effectiveness on anxiety. Further, the symptom reductions from pre to post in the “disruptive + emotional” group for the SCARED total anxiety and SCARED panic/somatic scores indicate only short-term effects in children with co-occurring anxiety. The effect did not persist in the long term as symptom levels slightly increased from post to follow-up, although they remained below the pre-training levels. The only large effect in both short- (pre to post) and long-term (pre to follow-up) can be seen in the SDQ emotional problems score showing a very strong symptom reduction in children with co-occurring emotional problems vs. a small symptom increase in children with DB only. This is probably due to the study design where the SDQ emotional problems score was used to split the sample into children with and without co-occurring emotional problems. The respective pre-training scores of all “disruptive + emotional” subjects were higher than the scores of all the “disruptive only” subjects and had a great potential for improvement. The score of the “disruptive only” group already was at the bottom of the scale and could thus only change to increase. Accordingly, the results of this interaction effect cannot be interpreted without bias and need to be re-examined in a sample that is independent of the measure’s pre-scores. Nevertheless, conclusions can be drawn from the SDQ emotional problems score results, which is why it was not excluded from the analyses. Children with co-occurring emotional problems exhibited great symptom reduction which can be seen in short- and long-term. However, SDQ emotional problems scores of these children clearly remained higher than the scores of children with DB only. Further, the majority of the “disruptive + emotional” group remained above the project’s prevention indication cut-off for emotional problems whereas only the minority of the “disruptive only” group rose above the SDQ emotional problems cut-off. In general, the hypothesis that the Baghira training is able to reduce emotional problems/anxiety and that children with co-occurring emotional problems show greater improvement cannot be confirmed. The results indicate that the Baghira training has, if any, only small and short-term effects on emotional problems/anxiety in children with DB and co-occurring emotional problems. In contrast, there is no evidence that children with only DB show an overall intervention effect on emotional problems/anxiety scores. Also, this study examined children with subthreshold psychological problems and symptom reduction could be greater/present with higher initial levels of emotional problems [58, 59]. However, the Baghira training was not able to reduce emotional problems to normal in the concerned children with co-occurring emotional problems and their emotional problems/anxiety scores did not approach those of the “disruptive only” group. Therefore, a prevention training targeting emotional problems/anxiety might still be indicated for these children after participation in the Baghira training. In this case, a separate well-established (prevention) program regarding emotional problems/anxiety seems appropriate. Apart from that, additional training sessions covering emotional problems/anxiety could be conducted. However, whether such additional content would have sufficient effects when administered as part of the Baghira training, following similar program routines has to be examined first. In fact, Levy et al. [40] found that additional content for managing child anger and aggression in a program targeting anxiety did not increase the symptom reduction in DB compared to the training without the additional content. Besides, given the very small to no improvement in emotional problems/anxiety in children with co-occurring emotional problems through the Baghira training content, several respective additional sessions would probably be needed to achieve reasonable prevention effects in these children. These additional sessions are not needed for children without co-occurring emotional problems and would take up unnecessary time and financial resources or children in need of additional content for emotional problems/anxiety have to be identified before conducting the additional sessions. Alternatively, specifically developed transdiagnostic prevention programs could be administered in children with co-occurring problems. Which of these options is most suitable should be examined in future research.

*Limitations*. Finally, there are some limitations to be mentioned. First, no alpha adjustment for multiple testing was administered. This leads to a potential overestimation of significant differences. Therefore, the results further support the conclusions of no differences in the effectiveness between groups and no or only small effects on emotional problems/anxiety. Second, this current non-randomized intervention study with no control group (neither untreated or usual care/differently treated nor unaffected/healthy subjects) does not allow for conclusions about the general efficacy and effectiveness of the Baghira training. Further, it is not able to confirm the causal relationship or to state how great the symptom reduction due to training is. However, the decreases in DB over time of the training and its stability over a six month period after the training in a naturalistic setting point into the direction of an effective intervention which is in line with Wettach et al. [25]. Third, training and questionnaire evaluations almost all took place during the Corona pandemic. On the one hand, this led to adjustments in the Baghira training, e.g. smaller groups, exercises without physical contact. On the other hand, the pandemic itself had a great burden on mental health in children and parents [60]. Training effects might have been counteracted due to the greater distressed children and parents who might have had less capacity to support the child. Further, contact restrictions might have reduced opportunities for practicing the training content. However, these effects of the Corona pandemic on the results cannot be ascertained. Fourth, the analyzed data are based on questionnaire information from one parent (or caregiver) and whether the children themselves perceived changes through training was not considered. Studies of the used measures with self-report versions available (SDQ, SCARED) showed medium correlations between self- and parent-report [53, 61]. Hence, results could differ by source of information. Therefore, future studies should additionally consider self-report and clinician evaluation. Fifth, this study focused on prevention and accordingly compared the effectiveness of an indicated prevention program in children with subthreshold DB/emotional problems. Therefore, the results of this study cannot be generalized to intervention effectiveness in children with clinical levels of DB and emotional problems. Sixth, the study took place in Dresden, Germany and transferability of the results to other regions and cultures is limited. This should be considered in future research. In general, the sample was derived based on systematic screenings tied to health check-ups at pediatricians that have very high participation rates [62] and can thus be considered as representative for the German population (urban areas) of children aged 5–10 years. Although, families with low socioeconomic status as well as of non-German nationality might be underrepresented due to somewhat lower participation rates in the health check-ups [62]. However, study groups of the sample did not differ in these characteristics. Seventh, treatment fidelity was not assessed, however, trainers were trained on-site and were provided with a detailed and clearly structured manual, and were regularly offered free supervision by a psychological psychotherapist.

## Conclusions

In sum, results do not evidence that children with DB and co-occurring emotional problems profited differently from the child-centered indicated group prevention program as children with DB only. The effectiveness of the indicated prevention program was not evidenced to be dependent of the pre-training anxiety. Assignment to a specific kind of DB training in case of co-occurring emotional problems/anxiety seems not necessary. The Baghira training had little to no proven effect on co-occurring emotional problems and anxiety except for the SDQ emotional problems score. In most cases, the indication for prevention regarding emotional problems/anxiety seemed to remain after the Baghira training in children with co-occurring emotional problems. A respective adapted or second training to target emotional problems is therefore advisable for these children. Further research is needed to examine whether children with co-occurring emotional problems actually exhibit symptom reduction in some emotional problems/anxiety measures through the Baghira training and whether the large effect on the SDQ emotional problems score is due to the study design.

## Data Availability

The data supporting the findings of this study are available from the senior author upon reasonable request.

## Footnotes

1 The regular routine pediatric health check-ups in Germany are called “U-Untersuchung” (Englisch “U-examination”) and are consecutively numbered from U1 (immediately after birth) to U11 (9 to 10 year olds).

## Abbreviations

Baghira training: Indicated group program for children with disruptive behavior problems, adapted from the Baghira group training
CBCL: Child Behavior Checklist
DB: Disruptive behavior
DISYPS: Diagnostic System for Psychiatric Disorders III
FBB-SSV: Parent Rating Scale for Oppositional Defiant Disorder and Conduct Disorder [German: Fremdbeurteilungsbogen für Störungen des Sozialverhaltens]
M: Mean
ODD: Oppositional Defiant Disorder
PCIT: Parent-child interaction therapy
Pre-anxiety: Pre-training SCARED total anxiety score
SCARED: Screen for Child Anxiety Related Emotional Disorders
SD: Standard deviation
SDQ: Strengths and Difficulties Questionnaire
Tiger training: Indicated group program for shy and anxious children “Mutig werden mit Til Tiger” (Becoming brave with Til Tiger)
U9/U10/U11: Regular routine pediatric health check-ups in Germany U-Untersuchung 9/10/11
TUD: TUD Dresden University of Technology
